# The Effect of Intra-Abdominal Hypertension Incorporating Severe Acute Pancreatitis in a Porcine Model

**DOI:** 10.1371/journal.pone.0033125

**Published:** 2012-03-05

**Authors:** Lu Ke, Zhi-hui Tong, Hai-bin Ni, Wei-wei Ding, Jia-kui Sun, Wei-qin Li, Ning Li, Jie-shou Li

**Affiliations:** Department of General Surgery, Jinling Hospital, Nanjing University, School of Medicine, Nanjing, China; Karolinska Institutet, Sweden

## Abstract

**Introduction:**

Abdominal compartment syndrome (ACS) and intra abdominal hypertension(IAH) are common clinical findings in patients with severe acute pancreatitis(SAP). It is thought that an increased intra abdominal pressure(IAP) is associated with poor prognosis in SAP patients. But the detailed effect of IAH/ACS on different organ system is not clear. The aim of this study was to assess the effect of SAP combined with IAH on hemodynamics, systemic oxygenation, and organ damage in a 12 h lasting porcine model.

**Measurements and Methods:**

Following baseline registrations, a total of 30 animals were divided into 5 groups (6 animals in each group): SAP+IAP30 group, SAP+IAP20 group, SAP group, IAP30 group(sham-operated but without SAP) and sham-operated group. We used a N_2_ pneumoperitoneum to induce different levels of IAH and retrograde intra-ductal infusion of sodium taurocholate to induce SAP. The investigation period was 12 h. Hemodynamic parameters (CO, HR, MAP, CVP), urine output, oxygenation parameters(e.g., S_v_O_2_, PO_2_, PaCO_2_), peak inspiratory pressure, as well as serum parameters (e.g., ALT, amylase, lactate, creatinine) were recorded. Histological examination of liver, intestine, pancreas, and lung was performed.

**Main Results:**

Cardiac output significantly decreased in the SAP+IAH animals compared with other groups. Furthermore, AST, creatinine, SUN and lactate showed similar increasing tendency paralleled with profoundly decrease in S_v_O_2_. The histopathological analyses also revealed higher grade injury of liver, intestine, pancreas and lung in the SAP+IAH groups. However, few differences were found between the two SAP+IAH groups with different levels of IAP.

**Conclusions:**

Our newly developed porcine SAP+IAH model demonstrated that there were remarkable effects on global hemodynamics, oxygenation and organ function in response to sustained IAH of 12 h combined with SAP. Moreover, our model should be helpful to study the mechanisms of IAH/ACS-induced exacerbation and to optimize the treatment strategies for counteracting the development of organ dysfunction.

## Introduction

Abdominal compartment syndrome (ACS) and intra-abdominal hypertension (IAH) may occur in many clinical situations such as burns, sepsis, bowel obstruction or peritonitis and have been recognized as a cause of organ dysfunction in critically ill patients [1], [2], [3]. The World Society of Abdominal Compartment Syndrome(WSACS) had reviewed the literature and reached a consensus about the definition of IAH and ACS in 2006 [4].

A tense abdomen is a common clinical finding in patients with severe acute pancreatitis(SAP), but the first report of the association of IAH and pancreatitis was only published in 2002 [5].After that, the association between IAH and SAP was confirmed repeatedly in several studies, Rosas et al suggested that the maximum intra abdominal pressure(IAP) was a prognostic marker of the evolution and complications of acute pancreatitis [6]. Al-Bahrani et al found that IAP correlated with the severity of organ failure, and a high admission IAP was associated with prolonged intensive care stay [7]. De Waele et al described a high incidence of IAH in patients admitted to the ICU because of SAP and IAH was associated with a high occurrence rate of organ dysfunction [8].

To our knowledge, there is no uniform consensus on the treatment of IAH/ACS in SAP patients and the detailed effects of IAH/ACS on different organ system have not been fully understood. Animal research is required to solve some of these problems regarding IAH/ACS over SAP because clinical prospective randomized studies are relatively difficult to perform. In the present study, the negative impact of SAP and IAH alone in a porcine model was investigated. Furthermore, the potential synergistic effects of different levels of IAH in pigs with SAP were also studied. Using this newly developed porcine model, we aimed to evaluate whether an IAP of 30 mmHg or 20 mmHg in combination with SAP would impact systemic hemodynamic responses and impair integrity and function of several organ systems comparable with the conditions in humans.

## Materials and Methods

### Animals

Thirty domestic female pigs aged approximately 3–6 months with a mean body weight of 26.0±2.2 kg(mean ± SD) were included in this study. Experiments were conducted in accordance with the Chinese legislation on protection of animals and “Principles of laboratory animal care” (NIH publication No. 85-23,revised 1985) and approved by the Animal Care and Use Committee of the Jinling hospital, and by the ethical committee of the School of Medicine of Nanjing University.

### Anesthesia and Hemodynamic Monitoring

All experimental animals were food deprived for 12 hours but had free access to water before the commencement of the procedure. After premedication with intramuscular ketamine 10 mg/kg and atropine 0.02 mg/kg, the animal was placed on the surgical table. General anesthesia was induced and maintained with ketamine(1 mg/kg/hr) and propofol(3 mg/kg/hr) via an ear vein. The animals were mechanically ventilated (Vela, Viasys Healthcare, San Diego, USA) volume cycled with a tidal volume of 8 mL/kg, an inspiratory oxygen concentration of 21%, a respiratory rate of 20/min and a positive end expiratory pressure of 2 cm H_2_O. These parameters were kept unchanged during the experiment. A central venous catheter in the left internal jugular vein was used for the continuous infusion of fluids and for measuring central venous pressure (CVP). Arterial blood samples were drawn from a catheter in the right femoral artery, which also monitored the arterial pressure and the heart rate(HR). A 7.0 F balloon-tipped Swan-Ganz fiberoptic pulmonary artery catheter (Edwards Lifesciences) was placed in the pulmonary artery, cardiac output(CO) measurements and mixed venous oxygen saturation (S_v_O_2_) were recorded on a monitor (Vigilance, Edwards Lifesciences, USA). Rectal temperature was kept 36°C to 38°C throughout the experiment by a heating pad. Urine output (UO) was recorded via a suprapubic catheterization.

### Surgical Procedure

After 1 h of steady-state phase, the animals were divided into the following 5 groups: SAP+30 mmHg IAP group(SAP+IAP30), SAP+20 mmHg IAP group(SAP+IAP20), SAP group(SAP), sham-operated+30 mmHg IAP group(IAP30) and sham-operated group(sham) and measurements of baseline values were performed, then all animals underwent a midline laparotomy. SAP was induced in 18 of 30(SAP+IAP30, SAP+IAP20 and SAP groups) by pressure-controlled(100 mmHg), intra-ductal infusion of sodium taurocholate (5%, 1 ml/kg BW, Sigma Chemical, St. Louis, MO, USA) and trypsin (7500 BAEE/kg BW, Difco Chemical, Detroit, MI, USA). The other 12 animals(sham and IAP30 groups) were intra-ductal retrograde infused normal saline(1 ml/kg BW) instead of sodium taurocholate and trypsin. After induction of pancreatitis, the incision was closed with a blanket suture, including the peritoneal sheath, all muscle layers and the fascia.

Afterward, a laparoscopic port of 5 mm was placed in the left side of the abdominal wall to inflate N_2_ pneumoperitoneum. IAH was induced in 12 of 18 SAP animals and IAP was maintained at 20 mmHg(SAP+IAP20 group) and 30 mmHg(SAP+IAP30 group) in each group, respectively. In addition, IAP was elevated to 30 mmHg in 6 of 12 sham-operated animals(IAP30 group). IAP was maintained during examination using an automatically controlled insufflator(Stryker endoscopy, MI, USA) with a pressure range of 0–30 mmHg. The other 6 SAP (SAP group) and 6 sham-operated animals(sham group) stayed with an unchanged IAP during the experiment. Lactated Ringer's solution was infused at a rate of 2 mL/kg/h using an automatic infusion device (Braun, Melsungen, Germany). Whenever the mean arterial pressure (MAP) dropped below 65 mmHg, the continuous intravenous fluid substitution was increased to 5 mL/kg/h. With persisting hypotension, the fluid substitution could be elevated to a maximum of 10 mL/kg/h, and in combination with dopamine infused with a maximum rate of 2 mg/kg/h. After 12 h, the animals were sacrificed by an overdose of potassium chloride, and histological specimens were taken from the pancreas, liver, small intestine, and lung following a standardized protocol.

### Hemodynamic and biochemical parameters

During that examination, MAP, CVP, and peak inspiratory pressure(PIP) were recorded in all groups just prior to pancreatitis induction (baseline) and every 3 h thereafter until the end of the experiment, while the UO was recorded every 2 hours. CO measurements and mixed venous oxygen saturation (S_v_O_2_) were recorded on a monitor (Vigilance, Edwards Lifesciences) at the same time via the Swan-Ganz fiberoptic catheter. PaO_2_, PaCO_2_, pH, base excess(BE) and serum lactate were measured by arterial blood-gas analysis every 3 h (ABL 510; Radiometer, Copenhagen, Denmark). Stroke volume (SV) was calculated as CO/HR, and systemic vascular resistance(SVR) was calculated as 80× (MAP-CVP)/CO. Abdominal perfusion pressure (APP) was calculated as MAP minus IAP. Intra vesical pressure (IVP) was used as IAP in non-IAH groups. Every 3 h in the experiments, additional samples were collected to determine the serum levels of sodium, potassium, glucose, aspartate aminotransferase (AST), alanine aminotransferase (ALT), bilirubin, amylase, serum urea nitrogen(SUN) and creatinine according to standard methods.

### Section protocol and histological examination

At the end of experiment, tissue specimens of lung, pancreas, liver and small intestine were promptly fixed in 4% phosphate-buffered formalin for 2 to 3 days and then embedded in paraffin. From the paraffin-embedded tissue blocks, 4-µm sections were cut and stained with hematoxylin and eosin (H&E) and examined light microscopically by a single pathologist blinded for the identities of the specimens.

The degree of microscopic injury was judged and scored based on different scales. As shown in Table 1–3, The lung was examined for pulmonary hyaline membrane, microthrombi, hemorrhage, edema, interstitial infiltration and atelectasis [9]. The pancreas was studied for edema, inflammation, vacuolization and necrosis [10]. The liver was assessed using the grade of congestion, vacuolization and necrosis [11]. Antimesenteric wedges of the distal ileum (10 cm from the ileocecal valve) were taken. The mucosal damage to the small bowel was evaluated on a grading scale from 0 to 8 as described previously by Park et al [12].

**Table 1:** Histopathological scoring criteria of lung.

Score	Hyaline membranes	Microthrombi	Edema	Interstitial infiltration	Atelectasis	Hemorrhage
0	Absent	Absent	Absent	Absent	Absent	Absent
1	Discrete	Few in small vessels	Discrete	Discrete	Small foci	Small foci
2	Moderate	Some	Moderate	Moderate	Larger foci	Larger foci
3	Severe	Many larger vessels	Severe	Severe	Diffuse	Diffuse

**Table 2 pone-0033125-t002:** Histological Scoring for Acute Pancreatitis.

Condition	Score	Description
Edema	0	Absent
	1	Diffuse expansion of interlobar septa
	2	1+ diffuse expansion of interlobular septa
	3	2+ diffuse expansion of interacinar septa
	4	3+ diffuse expansion of intercellular septa
Inflammation	0	Absent
	1	Around ductal margin
	2	In parenchyma (.50% of lobules)
	3	In parenchyma (51%–75% of lobules)
	4	In parenchyma (.75% of lobules)
Vacuolization	0	Absent
	1	Periductal (.5%)
	2	Focal (5%–20%)
	3	Diffuse (21%–50%)
	4	Severe (.50%)
Necrosis	0	Absent
	1	1–4 necrotic cells/HPF
	2	5–10 necrotic cells/HPF
	3	11–15 necrotic cells/HPF
	4	.16 necrotic cells/HPF

**Table 3:** Histological criteria for the assessment of liver damage.

Score	Congestion	Vacuolization	Necrosis
0	None	None	None
1	Minimal	Minimal	Single cell necrosis
2	Mild	Mild	−30%
3	Moderate	Moderate	−60%
4	Severe	Severe	>60%

### Statistical Analysis

All Data were analyzed using SPSS 13.0 for Windows (SPSS Inc., Chicago, IL, USA). Results were expressed as mean±SD. Normal distribution of the values was confirmed by Shapiro-Wilk test. Statistical analyses were performed with an analysis of variance for repeated measurements combined the Tukey post hoc test for changes within each group. Between-group analyses were performed by analysis of variance for factorial analysis with Bonferroni correction, the level of significance was adjusted according to Bonferroni. For the nonparametric results, the Mann-Whitney-U test was used. The Wilcoxon rank test was used to detect differences in histological injury score. P<0.05 was considered significant difference.

## Results

The body weight did not differ among the 5 groups at baseline (SAP+IAP30 26.0±1.4 kg; SAP+IAP20 25.7±2.7 kg; SAP 25.3±2.5 kg; IAP30 25.7±2.7 kg; sham 26.3±2.0 kg). One animal in SAP+IAP30 group died before the end of the experiment(11 h), while all the others survived the examination period. In SAP+IAH animals, an average of 2447±224 ml(SAP+IAP30) and 2294±314 ml(SAP+IAP20) of fluid volume were infused during the experiment, compared with 1421±193 mL in SAP group, 1039±233 ml in IAP30 group and 639±43 ml in sham group.

**Figure 1:**
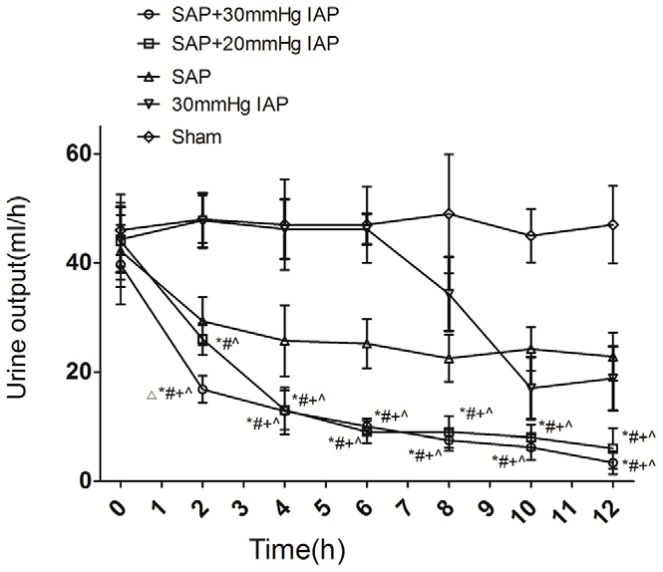
Urine output of SAP+IAH, SAP, IAH and sham groups during the experiment period (^▵^Significant difference vs. SAP+IAP20 group; ^*^Significant difference vs. sham group; ^#^ significant difference vs. IAP30 group; ^+^significant difference vs.SAP group; ^∧^significant difference vs. baseline values).

Under the condition of SAP combined with IAH, UO decreased similarly in both two groups(from 39.7±7.3 ml to 3.4±2.1 ml for IAP30 and from 44.0±7.1 to 26±2.9 ml for IAP20), although the SAP+IAP20 group had a bit higher UO during the first two hours after initiation. UO in SAP and IAP30 groups also displayed a mild decrease during the investigation, which is much less significant than two SAP+IAH groups (Figure 1).

### Hemodynamic Response

**Figure 2:**
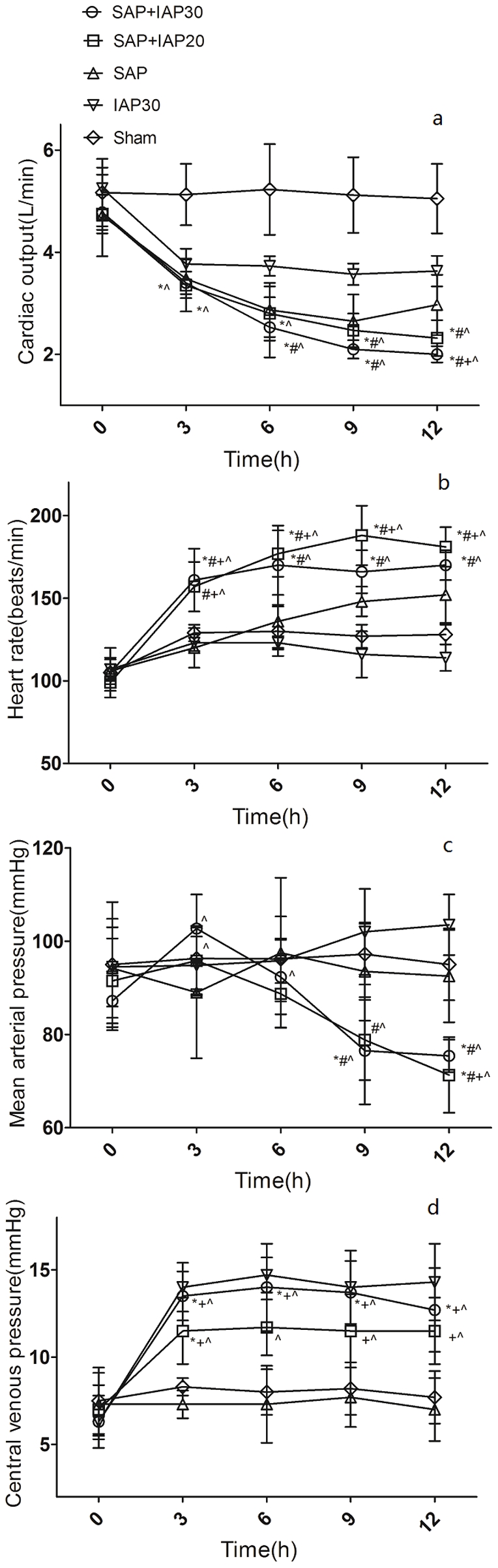
Hemodynamic responses of SAP+IAH, SAP, IAH and sham groups during the experiment period (*Significant difference vs. sham group; # significant difference vs. IAP30 group; ^+^significant difference vs.SAP group; ∧ significant difference vs. baseline values).

Despite more aggressive fluid resuscitation regimen, a significant decrease in MAP could be observed in both two SAP+IAH groups during the experiment, especially after 9 h(Figure 2c). Compared to groups without SAP, CO in all SAP animals decreased much more significantly after induction of pancreatitis, especially in groups combined with IAH (Figure 2a). This was paralleled by a profound fall in SV(Table 4) and a reciprocal increase in both HR(Figure 2b) and SVR(Table 4). Different levels of IAP lead to different increment of CVP in two SAP+IAH groups, although both seemed limited and no statistical difference could be detected, while CVP in SAP group did not change much over time(Figure 2d). In contrast, PIP increased nearly equal to the increase in IAP in all three groups with IAH and remained constant for the rest of the examination(Table 4).

**Table 4:** SV, SVR, APP and PIP during the experimental period.

Parameter	Group	0 h	3 h	6 h	9 h	12 h
SV(ml)	SAP+IAP30	45.5±4.4	21.7±3.0* # ∧	15.8±4.5* # ∧	13.0±1.4* # + ∧	11.8±1.6* # + ∧
	SAP+IAP20	48.1±4.3	21.7±5.2* # ∧	15.9±2.9* # ∧	13.1±1.0* # + ∧	12.8±1.5* # + ∧
	SAP	45.0±7.1	29.3±3.1	21.0±3.3	18.2±3.3	20.0±4.8
	IAP30	49.0±5.5	30.7±3.2	30.2±1.9	31.0±3.3	40.5±5.3
	Sham	49.2±6.7	39.5±4.2	40.2±5	40.5±5.3	39.5±4.4
SVR(dyne/s/cm^5^)	SAP+IAP30	1359±104	2077±270* ∧	2443±432* ∧	2339±319* ∧	2486±201* ∧
	SAP+IAP20	1430±191	2063±394* ∧	2286±549* ∧	2226±459* ∧	2113±462* ∧
	SAP	1497±228	1908±495	2618±727	2684±648	2397±576
	IAP30	1345±89	1725±191	1744±155	1980±249	1974±206
	Sham	1340±149	1386±134	1364±141	1406±143	1396±143
APP(mmHg)	SAP+IAP30	83.7±6.9	72.7±7.3∧	62.3±8.0* + ∧	46.5±11.5* # + ∧	45.3±3.5* # + ∧
	SAP+IAP20	88.2±8.9	75.0±8.0∧	67.5±7.5* + ∧	57.0±8.4* + ∧	50.5±7.5* # + ∧
	SAP	90.5±9.9	85.2±14.1	93.5±16.1	89.8±9.7	89.5±9.2
	IAP30	94.5±8.5	64.8±6.2	65.8±4.8	72±9.2	73.5±6.5
	Sham	91.3±13.4	91.3±6.1	93.0±9.1	93.0±7.3	91.5±7.9
PIP(cmH_2_O)	SAP+IAP30	17.8±2.3	50.0±3.0▵ * + ∧	50.0±2.8▵ * + ∧	49.2±2.4▵ * + ∧	50.0±1.0▵ * + ∧
	SAP+IAP20	17.3±2.8	40.5±1.6* # + ∧	39.3±1.8* + ∧	38.4±2.5* # + ∧	37.6±1.0* # + ∧
	SAP	17.0±2.5	16.8±0.8	16.7±1.6	18.3±1.6	22.8±2.4
	IAP30	18.3±2.7	47.3±3.4	45.3±4.3	47.2±3.7	45.5±3.4
	Sham	18.2±2.6	19.2±1.0	19.0±2.8	19.5±2.2	19.8±2.7

SV: stroke volume; SVR: systemic vascular resistance; APP: abdominal perfusion pressure; PIP: peak inspiratory pressure; SAP: severe acute pancreatitis; IAP: intra abdominal pressure;

▵ Significant difference vs. SAP+IAP20 group;

* Significant difference vs. sham group;

# significant difference vs. IAP30 group;

+ significant difference vs. SAP group;

∧ significant difference vs. baseline values.

The baseline IVP reading of SAP group was 3.7±1.6 mmHg, which was not different from all other groups(3.5±1.0 mmHg, 3.3±1.2 mmHg, 3.7±2.2 mmHg and 3.5±1.5 mmHg, respectively). During the investigation period, IVP was unaffected in SAP group which means SAP could not lead to IAH alone in a porcine model. The induction of SAP+IAH resulted in a remarkable drop of APP from 83.7±6.9 mmHg to 45.3±3.5 mmHg in SAP+IAP30 group and 88.2±8.9 mmHg to 50.5±7.5 mmHg in SAP+IAP20 group(Table 4). Although IAH alone could also lead to similar phenomenon, the tendency was much slower than the two SAP+IAH groups, while there were no significant changes in terms of APP in SAP and sham animals,

### Oxygen-derived Parameters

Both lactate level and S_v_O_2_ could be used to assess the balance of tissue oxygen delivery and consumption [13]. As shown in Table 5, lactate levels increased continuously throughout the examination period in two SAP+IAH groups. This was paralleled with a pronounced decrease in SvO_2_ from 71.0±2.9% to 40.2±5.9% for SAP+IAP30 group and 76.2±3.7% to 40.5±3.8% for SAP+IAP20 group. While in SAP and IAP30 groups, the lactate level showed an upbeat trend as well, but the level kept almost constant during the second half of observation. S_v_O_2_ in these two groups was also much higher than SAP+IAH animals over the entire experimental period, while the sham group kept stable in terms of both lactate and S_v_O_2_ levels during investigation.

**Table 5:** Oxygenation-derived parameters during the experimental period.

Parameter	Group	0 h	3 h	6 h	9 h	12 h
Lactate(mmol/L)	SAP+IAP30	0.95±0.16	1.97±0.28∧	2.87±0.79* ∧	3.60±0.80* # + ∧	3.80±0.94* + ∧
	SAP+IAP20	0.80±0.24	1.77±0.45∧	2.63±0.66* ∧	3.48±0.57* + ∧	3.92±0.81* + ∧
	SAP	0.90±0.14	1.38±0.31	1.95±0.42	2.05±0.24	2.08±0.31
	IAP30	1.03±0.33	1.68±0.33	2.05±0.28	2.30±0.38	2.58±0.48
	Sham	0.90±0.17	1.28±0.33	1.27±0.22	1.18±0.33	1.05±0.21
S_V_O_2_(%)	SAP+IAP30	71.0±2.9	51.1±2.2* # + ∧	42.0±5.7* # + ∧	40.5±5.6* # + ∧	40.2±5.9* # + ∧
	SAP+IAP20	76.2±3.7	58.9±3.8* ∧	48.5±7.2* # ∧	42.3±2.9* # + ∧	40.5±3.8* # + ∧
	SAP	71.4±3.5	64.6±8.2	60.8±8.1	52.9±5.1	53.6±5.5
	IAP30	73.1±5.0	65.8±7.9	64.9±7.4	60.5±6.6	61.0±9.9
	Sham	71.6±4.3	69.8±3.5	71.1±5.1	71.2±4.4	72.0±2.5
PO_2_(mmHg)	SAP+IAP30	86.0±6.1	77.9±5.8* ∧	72.9±4.4* ∧	74.6±2.6* # ∧	75.4±2.5* ∧
	SAP+IAP20	91.7±9.0	82.5±4.8∧	74.6±5.4* ∧	70.4±2.8* # + ∧	74.1±5.3* ∧
	SAP	91.5±11.9	89.8±14.2	87.9±11.0	86.1±9.7	83.7±10.6
	IAP30	91.0±4.1	89.8±7.0	84.0±7.7	83.3±5.4	81.2±2.9
	Sham	88.3±4.1	88.4±4.3	89.2±6.5	88.5±5.7	88.5±5.1
Base excess	SAP+IAP30	4.0±1.8	0.4±2.3* # ∧	−0.7±1.4* # + ∧	−1.8±2.0* # + ∧	−1.6±1.6* # + ∧
	SAP+IAP20	4.0±1.0	1.1±0.9# ∧	0.0±0.9* # + ∧	−1.1±1.2* # + ∧	−1.6±1.6* # + ∧
	SAP	4.5±1.2	3.6±1.3	4.2±1.3	3.3±1.1	2.9±1.2
	IAP30	3.4±1.1	4.0±0.9	3.8±1.3	2.6±1.2	2.4±1.4
	Sham	4.3±1.1	4.1±1.4	4.6±1.4	4.6±1.3	4.5±1.2

SAP: severe acute pancreatitis; IAP: intra abdominal pressure; SVO_2_: mixed venous oxygen saturation; PO_2_: partial pressure of oxygen;

* Significant difference vs. sham group;

# significant difference vs. IAP30 group;

+ significant difference vs.SAP group;

∧ significant difference vs. baseline values.

The level of arterial PO_2_ showed a slight decrease in both SAP and IAP30 groups and a more pronounced decrease in two SAP+IAH groups(Table 5). The blood gas analyses also showed that the arterial PaCO_2_ in all groups remained almost unchanged when compared with the baseline values. In all SAP+IAH animals, mild acidosis occurred with the pH decreasing slightly during the observation, while a concomitant reduction in BE could also be observed (Table 5). In contrast, the other three groups were almost not affected in terms of both pH and BE throughout the experiment.

### Biochemical Parameters

The plasma activity of amylase showed a noteworthy increase in all 18 SAP animals, but there's no statistical difference among these 3 groups(Table 6). In addition, Table 6 also shows that several commonly used clinical indicators of liver and kidney injury including AST, creatinine and SUN increased more remarkably in SAP+IAH groups when compared with SAP and IAH alone animals.

**Table 6:** Biochemical parameters during the experiment period.

Parameter	Group	0 h	3 h	6 h	9 h	12 h
Amylase(U/L)	SAP+IAP30	595±153	2877±841* # ∧	4665±901* # ∧	5773±1107* # ∧	6766±1945* # ∧
	SAP+IAP20	680±160	2923±680* # ∧	4690±847* # ∧	5472±1141* # ∧	6455±2099* # ∧
	SAP	709±153	2629±859	3882±1525	4428±1334	5195±1019
	IAP30	748±139	1400±373	1788±198	1953±194	2087±107
	Sham	680±144	1194±265	1392±344	1493±255	1488±194
AST(U/L)	SAP+IAP30	77±10	164±26* # ∧	213±28* # + ∧	262±44* # + ∧	279±34* # + ∧
	SAP+IAP20	57±12	153±27* # ∧	190±30* ∧	233±48* ∧	279±46* # + ∧
	SAP	86±15	119±22	145±25	160±24	168±29
	IAP30	80±19	99±14	141±18	163±18	191±12
	Sham	80±17	86±20	91±19	94±12	97±8
SUN(mmol/L)	SAP+IAP30	3.15±0.99	5.82±1.60∧	8.53±1.34* # + ∧	10.68±2.02* # + ∧	10.92±1.25* # ∧
	SAP+IAP20	2.57±0.48	4.48±1.09∧	7.70±1.48* # ∧	9.72±2.07* # + ∧	10.77±2.50* # ∧
	SAP	3.35±0.54	4.12±0.99	5.18±1.47	5.88±1.68	7.12±1.54
	IAP30	3.26±0.62	3.52±0.53	3.78±0.43	4.28±0.44	4.70±0.75
	Sham	3.33±0.73	3.80±0.64	4.08±0.52	3.88±0.67	4.10±0.85
Creatinine(µmol/L)	SAP+IAP30	74±24	151±22* # ∧	225±37* # ∧	287±25* # + ∧	314±29* # + ∧
	SAP+IAP20	72±12	148±25* # ∧	197±30* # ∧	241±33* # ∧	313±36* # + ∧
	SAP	76±18	131±33	177±35	205±28	226±243
	IAP30	67±11	92±11+	106±10	149±23	181±30
	Sham	77±18	90±18	96±20	99±15	105±15

SAP: severe acute pancreatitis; IAP: intra abdominal pressure; AST: Aspartate transaminase; SUN: serum urea nitrogen;

* Significant difference vs. sham group;

# significant difference vs. IAP30 group;

+ significant difference vs.SAP group;

∧ significant difference vs. baseline values.

### Histology

**Figure 3:**
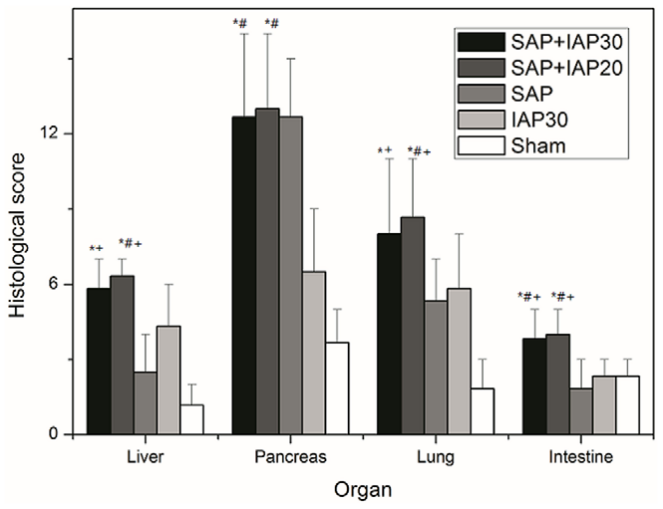
The comparisons of histopathology scores among five experimental groups (*Significant difference vs. sham group; # significant difference vs. IAP30 group; ^+^significant difference vs.SAP group).

**Figure 4:**
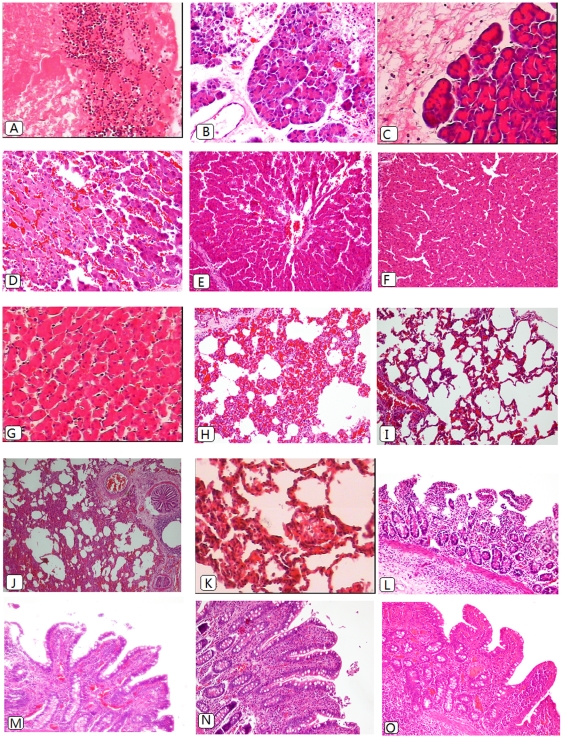
Histomorphological images of pancreas (A–C), liver (D–G),lung(H–K) and ileum (L–O) of SAP+IAH animals (A,D,H,L),SAP animals(E,I,M), IAP 30 mmHg animals(B,F,J,N) and sham-operated animals(C,G,K,O). Remnant necrotic tissue with a great number of inflammatory cells infiltration(A×200).Mild-to-moderate-grade edema and degenerative changes with very mild necrosis(B×200). Mild edema and inflammation without evidence of necrosis(C×200). Livers of the study groups (D×400) showed more severe congestion and necrosis when compared with the SAP group(E×200),while there were mild-to-moderate-grade congestion, vacuolization and leukocyte infiltration in IAP 30 mmHg group(F×200). Only minor changes could be observed in the sham controls (G, 400×). The lung of the SAP+IAH animals displayed interstitial infiltration, focal hemorrhage and atelectasis(H×200) while the specimen of SAP animal presented lower grade injury(I×200). The lung specimen of IAH alone animals displayed atelectasis as well, but only moderate hemorrhage and interstitial infiltration(J×200). Slight edema and infiltration were seen in sham animals(K×400). The small bowel specimen of the study group (L×200) displayed denuded villi. The specimen of SAP animals presented extended subepithelial space and lymph follicle hyperplasia(M×200), while in the IAH group, there was similar histological changes to the SAP group and also lymph follicle hyperplasia(N×200). In the sham control, only minor changes could be seen(O×200).

The histopathological results of the pancreas, liver, small bowel and lung are shown in Figure 3. Since the pathological presentations of SAP+IAP30 and SAP+IAP20 animals were similar, we only included typical graphs for both of these two groups. Histological analysis of pancreas tissue demonstrated severe necrosis in all SAP animals and in some of which no pancreatic issue could be seen (Figure 4A). The specimen of IAP30 animals presented degenerative changes with very mild necrosis (Figure 4B), while the sham-operated animals only presented mild edema and inflammation(Figure 4C)

The histological examination of the liver yielded high-grade intrasinusoidal congestion and mild-to-moderate necrosis in SAP+IAH animals(Figure 4D). In the SAP animals, there were only moderate congestion and minimal vacuolization(Figure 4E), while medium-grade congestion, vacuolization and leukocyte infiltration could be seen in IAH alone animals(Figure 4F). For the controls, only minor congestion was observed (Figure 4G). As shown in Figure 4H, the induction of SAP+IAH resulted in formation of moderate interstitial infiltrates, focal hemorrhage and large foci of atelectasis in the lower lobe as compared with lower grade damage absent of significant atelectasis in the SAP group(Figure 4I). As for IAH only animals, although there was formation of atelectasis as well, but the grade of hemorrhage and interstitial infiltration was lower (Figure 4J), while only mild edema and infiltration were seen in the sham controls(Figure 4K). There was no difference in terms of microthrombi and hyaline membranes.

Intestinal histopathology in SAP+IAH animals demonstrated significantly worse tissue injury than that in SAP animals (Figure 4L,M). In IAH alone animals, moderate mucosal damage such as extended subepithelial space was observed (Figure 4N). In the sham group, the villus and glands were almost normal and no inflammatory cell infiltration was observed in mucosal epithelial layer(Figure 4O).

## Discussion

No animal studies evaluating the effect of IAH in combination with SAP have been published yet. In this porcine model, we first described the effect of different levels of IAH incorporating SAP on systemic hemodynamics, oxygenation and organ functions in detail.

Not resembling human beings, pigs have broad space in abdominal cavity in addition to the good compliance of their abdominal wall so that SAP could not lead to IAH alone in a porcine model evidenced by constant IVP we observed in SAP group. Therefore, we developed a new animal model in which we applied pneumoperitoneum over a widely accepted SAP model, since it is easy to deploy and does not interfere with fluid status. As clinical and experimental studies have observed a peritoneal absorption of CO_2_ at an IAP of 15 mmHg and lead to mild acidosis [14], we chose Nitrogen(N_2_)-pneumoperitoneum because N_2_ is colorless, odorless, tasteless and generally inert. Previous studies showed the application of N_2_ in laparoscopy did not impact pH, cardiac output and mean arterial pressure. Although it may increase the risk of venous gas emboli due to its poor solubility in the blood, the incidence rate is still very low [15].

As different IAP could lead to different consequences, we selected two levels of IAP for different purposes. On the one hand, based on the known negative impact of 30 mmHg IAP in porcine models [16], [17], [18], we chose this relatively high pressure to mimic an extreme condition to detect “how bad could it be”. On the other hand, we also studied another IAP of 20 mmHg, which is of more clinical relevance and served as the diagnostic standard of ACS in the definitions established by WSACS [19]. However, few statistical differences were found between the two groups except the urine output during the initiative two hours and the PIPs, although the 20 mmHg animals seemed a bit less severe sick in terms of several parameters, especially in the first a few hours. The results indicate that the presence of an IAP of 20 mmHg or more in SAP patients could be very stringent and surgical decompression should be considered once ACS has been diagnosed.

Regarding the hemodynamic changes, we found there was a profound reduction in CO in all groups except sham group, but the reduction was much more pronounced in SAP+IAH animals during the experiment which was accompanied with the reduction of SV. Thus, the significant reduction of CO might be mainly due to a reduced SV, which in turn probably reflects reduced ventricular filling as illustrated by the relative low CVP(CVP corrected = CVP measured−IAP/2) [20] and extremely high HR which could also shorten the ventricular filling time. The relatively slight reduction of CO in IAH only animals correlated well with the results of previous investigations [16], [17], [21], [22] which indicated that SAP contributed a lot to the reduction of CO. The mechanisms underlying this phenomenon should include both systemic inflammatory response and third space fluid sequestration caused by SAP and significant increased intrapleural pressure and decreased venous return caused by IAH. Furthermore, the induction of IAH led to oliguria(IAH group) or even anuria(SAP+IAH group) which suggested that extremely elevated IAP could alter renal function significantly even without SAP. The sharply increased PIP in all animals with IAH was caused by the increased IAP via the trans-diaphragmatical pressure propagation and could have resulted in a reduction of total lung capacity, functional residual capacity [23] accompanied with an increased afterload in pulmonary circulation which might also contribute to the reduction of SV.

Previous studies have shown the deleterious effects of both IAH and SAP on pulmonary function and systemic oxygenation [17], [21], [22], [24]. In this study, minute ventilation was constant because of the applied volume-cycled ventilation mode. Dissolved oxygen (as expressed by PO_2_) decreased substantially in two SAP+IAH groups and slightly in both SAP and IAH groups. The results are in consistent with Schachtrupp et al's study which detected no significant changes of arterial PO_2_ under the condition of ACS(30 mmHg) alone during 24 hours when ventilated using an inspiratory oxygen concentration of 25% [21]. Therefore, we could infer that SAP itself has substantial impact to systemic oxygenation through diffuse alveolar damage, microvascular injury and influx of inflammatory mediators. The animals in SAP+IAH group presented mild acidosis though PCO_2_ stayed constant during the investigation. Therefore this should be mostly due to metabolic mechanisms evidenced by significantly decreased BE in SAP+IAH animals. The most likely mechanisms behind the decreased BE would be the prominent increase of lactate level and renal dysfunction illustrated by increased serum creatinine and urea nitrogen levels.

Both experimental [25] and clinical studies [26], [27] have pointed out that tissue hypoxia, characterized by supply-dependent oxygen consumption, as a cause of increased lactate levels which was thought to be associated with significant morbidity and mortality. Besides that, the Surviving Sepsis Campaign guidelines recommend the use of central venous oxygen saturation (ScvO_2_) or mixed venous oxygen saturation(S_V_O_2_) to assess the balance of tissue oxygen delivery and consumption. Our data on lactate level and S_V_O_2_ suggested that SAP+IAH animals suffered much more severe peripheral tissue hypoxic when compared with SAP or IAH alone pigs no matter which pressure was applied.

The aggravation of distal organs dysfunction is the mostly referred consequence when IAH is combined with SAP, but whether IAH could aggravate the injury of pancreas was not clear. Otto et al indicated that 6 h and 12 h of IAH could lead to light microscopical and ultrastructural changes in pigs comparable to pancreatitis in humans which means IAH could lead to mild pancreatitis alone [18]. Our data suggested the activity of amylase which is used as a hallmark of pancreatitis was not different between SAP animals with or without IAH. Additionally, the application of IAH did not significantly alter the histological score of pancreas in SAP animals. That might be due to SAP itself could cause severe histological damage to the pancreas evidenced by extremely high score for pancreatic injury in all SAP animals. Regarding the lung, light microscopic findings indicated animals in all groups except sham group presented focal or diffuse alveolar damage, represented by partial thickened or ruptured alveolar septum, focal hemorrhage and interstitial inflammatory cell infiltration in alveolar spaces. Formation of atelectasis in the lower lobes of the lung in SAP+IAH and IAH alone animals appeared to be secondary to an increased IAP, leading to diaphragmatic eventration, and compression of the lung tissue [21].

Several biochemical parameters including ALT,SUN and creatinine increased significantly in all groups except sham animals, but the ascending tendency was much faster in SAP+IAH groups. These results suggested more severe organ damage in SAP+IAH animals, which were consistent with the histological results. There have been both clinical and experimental studies about intestinal injury under the condition of IAH/ACS or SAP [28], [29], [30], [31], [32], [33], [34], [35], but whether the combination of these two will aggravate the intestinal injury has not yet been evaluated. Our data strongly suggested that the occurrence of SAP+IAH could significantly increase the severity of intestinal injury which means more bacterial translocation and worse motility.

In this study, we successfully described the “natural course” of different levels of IAH in combination with SAP, and our data suggested that 12 h of consistent IAH could lead to lethal impairment of multiple organ systems, therefore more aggressive interventions such as operative decompression may be necessary in these patients. Mentula et al recently reported that early surgical decompression was associated with reduced mortality in SAP patients [36]. But more details need to be addressed to optimize the treatment and avoid unnecessary decompressive laparotomy since “open abdomen” is not easy to manage and could result in severe and lethal complications. Our porcine model will be a good platform for these studies with different purposes, such as searching for the optima timing(at what time) and the indicator(at what pressure) of decompression, what kind of approach could lead to best prognosis,etc.

Several limitations of our study warrant discussion. A possible drawback of this study could be the relatively short period compared to earlier studies [17], [21], [22] which mostly chose 24 h. A duration of 12 h was chosen because we found that most animals will otherwise die before the end of experiment under the condition of SAP+IAH,but it led to obviously slighter histological organ damage in SAP animals than earlier studies [37]. In addition, IAH in our experiment was caused by N_2_-derived pressure increase instead of pancreatitis itself which may bring in some uncertainty about whether this model could reflect the clinical condition.

In conclusion, the induction of a porcine SAP+IAH model worked well for studies of hemodynamics, pulmonary function, systemic oxygenation and organ damage. The results indicated that there were remarkable effects on global hemodynamics and organ functions in response to sustained IAH of 12 h combined with SAP resembling the conditions defined in humans. Additionally, our model should be helpful to study the mechanisms of IAH-induced exacerbation and to evaluate novel strategies for counteracting the development of organ dysfunction after initiation of IAH with SAP.
